# Learning and memory disabilities in IUGR babies: Functional and molecular analysis in a rat model

**DOI:** 10.1002/brb3.631

**Published:** 2017-02-07

**Authors:** Marta Camprubí Camprubí, Rafel Balada Caballé, Juan A. Ortega Cano, Maria de los Angeles Ortega de la Torre, Cristina Duran Fernández‐Feijoo, Montserrat Girabent‐Farrés, Josep Figueras‐Aloy, Xavier Krauel, Soledad Alcántara

**Affiliations:** ^1^ Neonatology ServiceSant Joan de DéuBCNatalHospital Sant Joan de Déu i ClínicUniversity of BarcelonaBarcelonaSpain; ^2^ Department of Pathology and Experimental TherapeuticsSchool of MedicineUniversity of BarcelonaBarcelonaSpain; ^3^ Department of Molecular Biology and Biochemical EngineeringUniversity Pablo de Olavide – CABIMER – CIBERDEMSevillaSpain; ^4^ Pediatrics ServiceHospital Álvaro CunqueiroVigoSpain; ^5^ Universitat Internacional de CatalunyaSant Cugat del VallèsBarcelonaSpain; ^6^ Present address: Department of NeurologyFeinberg School of MedicineNorthwestern UniversityChicagoIL60611USA

**Keywords:** cephalization index, intrauterine growth restriction, learning, placental insufficiency, spatial memory, synaptic plasticity

## Abstract

**Introduction:**

Intrauterine growth restriction (IUGR) is the failure of the fetus to achieve its inherent growth potential, and it has frequently been associated with neurodevelopmental problems in childhood. Neurological disorders are mostly associated with IUGR babies with an abnormally high cephalization index (CI) and a brain sparing effect. However, a similar correlation has never been demonstrated in an animal model. The aim of this study was to determine the correlations between CI, functional deficits in learning and memory and alterations in synaptic proteins in a rat model of IUGR.

**Methods:**

Utero‐placental insufficiency was induced by meso‐ovarian vessel cauterization (CMO) in pregnant rats at embryonic day 17 (E17). Learning performance in an aquatic learning test was evaluated 25 days after birth and during 10 days. Some synaptic proteins were analyzed (PSD95, Synaptophysin) by Western blot and immunohistochemistry.

**Results:**

Placental insufficiency in CMO pups was associated with spatial memory deficits, which are correlated with a CI above the normal range. CMO pups presented altered levels of synaptic proteins PSD95 and synaptophysin in the hippocampus.

**Conclusions:**

The results of this study suggest that learning disabilities may be associated with altered development of excitatory neurotransmission and synaptic plasticity. Although interspecific differences in fetal response to placental insufficiency should be taken into account, the translation of these data to humans suggest that both IUGR babies and babies with a normal birth weight but with intrauterine Doppler alterations and abnormal CI should be closely followed to detect neurodevelopmental alterations during the postnatal period.

## 1 Introduction

Intrauterine growth restriction (IUGR) is the failure of the fetus to achieve its inherent growth potential and can be due to maternal or fetal problems (Goldenberg et al., 1989). The incidence of IUGR in developed countries is around 3–7% of the total population, but may vary depending on the criteria used (Romo et al., 2009). The degrees and definitions of IUGR differ, but recent diagnostic criteria consider fetuses to be truly IUGR when the estimated weight is less than the 10th percentile for gestational age and on presenting an abnormal fetal Doppler ultrasound. Severe IUGR is defined as weight below the 3rd percentile (Figueras & Gardosi, 2011; Zhang, Merialdi, Platt, & Kramer, 2010). However, according to the growth standards applied, a number of babies may be considered growth restricted (not achieving their potential genetic growth) but not IUGR, because their birth weight (BW) is within the 10th percentile or above and considered in the normal range.

Children born with IUGR are predisposed to long‐term cognitive and neurodevelopmental impairment that is associated with learning difficulties and decreased academic performance (Arcangeli, Thilaganathan, Hooper, Khan, & Bhide, 2012; de Bie, Oostrom, & Delemarre‐van de Waal, 2010). Longitudinal studies on IUGR babies have recently identified the cephalization index (CI = head circumference/BW) as one of the best perinatal parameters for the prediction of neurodevelopmental outcome (Geva, Eshel, Leitner, Fattal‐Valevski, & Harel, 2006). A high CI in IUGR babies seems to be indicative of intrauterine blood flow redistribution, also known as the *brain sparing* effect, and is strongly correlated with a reduction in IQ, difficulties in creative problem solving, deficits in attention and executive functions, and alterations in visuomotor organization (Flood et al., 2014; Van den Broek, Kok, Houtzager, & Scherjon, 2010).

The underlying mechanisms of neurologic impairment in IUGR remain unclear, although some authors have suggested that they may be associated with structural brain alterations. A significant reduction in intracranial volume and cortical gray matter have been reported in IUGR babies, and some authors have suggested that a selective cortical vulnerability may be involved (Eixarch et al., 2012).

In the same line, neuroimaging and histopathological studies of the IUGR brain in infants and animal models have shown that one of the areas most affected is the hippocampus (Dieni & Rees, 2003; Lister et al., 2005; Lodygensky et al., 2008). The hippocampus is a structure involved in codification, storage, and recovery mechanisms related to learning and memory processes (Vann & Albasser, 2011). In the hippocampus, Cornus Ammonis 1, 3 (CA1, CA3) and dentate gyrus are the most vulnerable regions to hypoxia‐ischemia (HI), an inherent condition in utero‐placental insufficiency (Hsiao & Patterson, 2012). The main structural changes described include a reduction in cell number and white matter content and altered cellular composition and dendrite morphology (Dieni & Rees, 2003; Fung et al., 2012; Mallard, Loeliger, Copolov, & Rees, 2000). All these changes have also been associated with modifications in the gene expression of neurotrophic factors, glutamate neurotransmitter receptors, and synaptic proteins that eventually lead to important synaptic alterations (Chen et al., 2007; Dieni & Rees, 2005; Schober et al., 2009; Sommer et al., 2010).

The aim of this study was to assess the effect of utero‐placental insufficiency on learning and memory processes. We used a CMO animal model of IUGR (Camprubi et al., 2009) to determine the correlation between functional deficits in learning and memory and synaptic proteins expression in the hippocampus. Moreover, we also studied whether the CI might be a good marker to identify patients with a brain‐sparing effect despite their BW being within the normal range.

## 2 Materials and methods

### 2.1 CMO procedure and anthropometric analysis

The studies were designed to minimize the number of animals used in the procedure. All the animal protocols were approved by the Institutional Animal Care and Use Committee in accordance with Spanish and European Union regulations.

Thirty‐two pregnant Wistar rats (Harlam Laboratories) were maintained in a 12 h‐light/dark cycle with *ad libitum* access to standard rodent diet and water. The CMO procedure was performed in pregnant rats at embryonic day 17 (E17) (*n *= 17). Briefly, under general anesthesia with isofluorane 2.5% dams underwent laparotomy and two meso‐ovarian vessels located in the borders between the upper and medium and the medium and lower thirds of the corresponding horn were cauterized. This procedure has been validated as a placental insufficiency induction method (Camprubi et al., 2009). Control animals underwent identical anesthesia and both uterine horns and their vessels were exposed and examined with no further manipulation (*n *= 15). The CMO model produces more uniform ischemia throughout the uterus and therefore affects all the implanted fetuses more homogeneously than the uterine artery ligation method described by Wigglesworth (1964). In CMO model BW was not dependent on the position of the fetuses and was quite homogeneous, with a similar proportion of pups <10th centile in each litter (Wigglesworth 1964; Camprubí et al., 2009).

The rats were allowed to deliver spontaneously and the pups were born between days 21 and 22 of gestation. To avoid rejection by the mother, newborn pups were counted and their BW recorded on the second day of life (P2) (accuracy: 0.1 g; PB 3001‐L Mettler Toledo).

**Figure 1:**
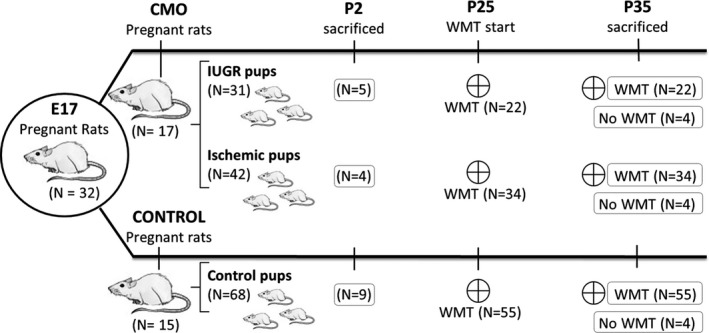
Graphical scheme of the experimental groups and procedures. WMT: water maze test

The animals were then divided into three experimental groups with both sexes being similarly represented. Pups born to sham‐operated mothers were considered as controls (*n *= 68). According to human diagnostic criteria, pups born to CMO‐operated mothers were divided into two different experimental groups: the animals most affected were denominated the *IUGR group* which was composed of pups with a BW below the 10th percentile (<5.07 g, *n *= 31); and those with mild involvement but with a normal BW (≥10th percentile) were denominated *ischemic* (≥5.07 g, *n *= 42). To ensure a homogeneous milk supply throughout the litter, a maximum of four rat pups were allowed per mother. In the case of controls, only the pups with a normal BW were included in the study. A scheme of the number and procedures performed in the animals studied is shown in Figure 1.

On day 35 (P35), the rats were weighed and the growth velocity was calculated as:

[Weight atP35 (g)−weight at P2 (g)]/33(days).

To evaluate the *brain‐sparing effect*, nine controls, four ischemic and five IUGR pups were sacrificed and analyzed at P2 and 16 controls, 15 ischemic and 14 IUGR pups were studied at P35. The brains were rapidly removed and weighed. Taking into account the difficulty and low accuracy of head circumference measures in rats, the CI (calculated in humans as CI = head circumference × 100 /BW (g)) was calculated using the weight of the brain. In these animals a modified version of the human CI was calculated as CI = brain weight (mg) × 100 / BW (mg). This modified version of the CI reflects the brain/BW ratio, expressed as a percentage.

### 2.2 Water maze test

Spatial memory analysis was carried out according to a version of the Morris’ water maze (MWT) pool modified for small animals (63 cm long, 43 cm wide and 35 cm high), with the water temperature set at 22–23°C and made opaque by latex suspension (Morris, 1984). The escape latency (EL), defined as the time taken to reach the platform, was measured during each trial as an indicator of learning. On postnatal day 25, the pups of each group (55 controls, 34 ischemic and 22 IUGR) were trained for 10 consecutive days and then sacrificed at day 35. The CI of these animals was calculated and its influence on the learning curve analyzed.

### 2.3 Western blots (WB)

As shown in Figure 1, a group of 50 rats (14 IUGR, 15 ischemic and 16 controls) were trained in the MWT from P25 to P35 (trained animals), four animals from each group (*n *= 12) were not trained (untrained animals). The rats were sacrificed at day 35 by decapitation and their brains were rapidly removed, weighed and processed for WB analysis.

Protein extracts from the hippocampus were separated by SDS‐PAGE and electro‐transferred to a nitrocellulose membrane. Membranes were blocked with 5% nonfat dry milk in Tris‐buffered saline and incubated first with primary antibodies overnight at 4°C (1:2000, PSD95; Abcam, Cambridge, UK; 1:1000; Synaptophysin, Dako, Glostrup, Denmark; 1:10,000, goat anti‐Actin, 1:10,000; Santa Cruz Biotechnology, San Diego, CA, USA), and then with their corresponding secondary HRP‐conjugated antibodies (1:5000, Santa Cruz Biotechnology). The protein signal was detected using the ECL chemiluminescent system (Amersham, Buckinghamshire, UK). Quantification of the labeling intensity in micrographs and densitometry analysis for WB bands was carried out using ImageJ software (Rasband, W.S., ImageJ, U. S. National Institutes of Health, Bethesda, MD, USA, http://rsb.info.nih.gov/ij/, 1997–2009). *Histogram* and *gel* tools were used to analyze confocal micrographs and WB bands, respectively. All trained (14 IUGR, 15 ischemic and 16 controls) and untrained (4 per group) animals were analyzed, loading 3–4 animals per group and gel, followed by a comparative study.

### 2.4 Histological and immunohistochemistry analysis

For histological and immunohistochemistry analysis, trained rats (8 IUGR, 19 ischemic and 39 controls) were killed by anesthetic overdose and transcardially perfused with 4% paraformaldehyde (PFA). Dissected brains were postfixed for 6 h in 4% PFA, cryoprotected and kept frozen. Coronal sections of 40‐μm thick were collected in a cryoprotective solution (40% 0.1 M phosphate buffer, 30% glycerol, and 30% ethylene glycol), and stored at −30°C for further use.

For broad histological analysis tissue sections were mounted on polylysine coated slices and stained with Hematoxylin–Eosin (H‐E). Immunohistochemistry was performed in tissue sections blocked with 10% methanol in PBS for 15 min followed by 0.4 triton X‐100 PBS + 10% goat/horse normal serum (Gibco‐Life Technologies refs. 16210–072/16050–122) for 2 hr and subsequently incubated with primary antibodies at 4°C overnight. The sections were then incubated with biotin‐conjugated secondary antibodies (1:200, Vector, Burlingame, CA, USA) for 1 hr and subsequently with a Streptavidin‐peroxidase complex (1:400; Amersham) for 2 hr at room temperature. The enzymatic reaction was developed with diaminobenzidine (DAB, ref. D123384‐SG; Sigma‐Aldrich Co, Sant Louis, MO, USA) and the sections were dehydrated and coverslipped with Eukitt (Panreac, Barcelona, Spain). Primary antibodies against the following proteins were used: GFAP (1:1000, ref. Z0334 Dako), NeuN (1:500, ref. MAB377, Millipore, Tamecula, CA, USA) PSD95 (1:1000, ref ab18258, Abcam), Synaptophysin (1:1000, ref IR776, Dako).

Micrographs were captured with a light microscope Nikon Eclipse 800 (Nikon, Tokio, Japan) and assembled in Adobe Photoshop (v. 7.0), with the same adjustments for contrast, brightness, and color balance to obtain optimum visual reproduction of the data. The number of CA1 pyramidal neurons was counted at the level of the dorsal hippocampus in 400× micrographs of NeuN‐stained sections with the aid of ImageJ software. Four serial pictures taken 300 microns apart of three animals per group were used.

### 2.5 Data analysis

The daily EL was averaged from four training trials and analyzed using multifactor ANOVA (time, group and sex) and the Fisher's least significant difference (LSD) test. ANCOVA test was used to analyze the influence of the CI and Birth weight on the learning process. Differences in cell number, CI, growth velocity, and synaptic protein expression among the three groups were calculated using one‐way ANOVA and the Fisher's least significant difference (LSD) test. The statistical analysis was performed using Statgraphics Centurion program.

## 3 Results

### 3.1 Birth weight and growth rates of the CMO and control groups

According to experimental manipulation and BW, the pups were divided into one control group and two CMO groups: ischemic and IUGR; depending on their birth weight. Males and females were equally represented in each group (*p* = .73).

Some of the animals were killed at day 2 (see Table 1 for anthropometric data).

**Table 1:** The anthropometric parameters of newborn rats

	Control *N *= 9	Ischemic *N *= 4	IUGR *N *= 5
Birth weight (P2) (g)	6.13 ± 0.39	**5.66 ± 0.27** *	**4.45 ± 0.13** **,##
Brain weight (P2) (g)	0.24 ± 0.02	**0.27 ± 0.01** **	0.26 ± 0.01
Cephalization index at day 2	3.99 ± 0.32	**4.86 ± 0.23** **	**5.87 ± 0.07** **,##

IUGR, Intrauterine growth restriction. In the table, Birth weight , brain weight and cephalitzation index at day two is presented.

Each group belongs to at least two different mothers. Data are indicated as mean ± SD.

Significant differences compared to controls **p < .01, *p < .05; to Ischemic rats ##*p* < .01.

The remaining animals were killed at P35 and some were studied by WB, with the brains of these animals being extracted in fresh and weighed (see Table 2 for anthropometric data). The other animals, used for histology, were perfused and their brains were not weighed. As reported previously (Camprubi et al., 2009), the CI of the newborn IUGR pups was significantly higher than that of the control animals (*p* < .01). Surprisingly, although the BW of the ischemic pups was within the normal range (≥10th percentile), their CI was significantly higher than that of the controls, but lower than the IUGR group (*p* < .01, Table 1). Anthropometric parameters were also recorded in animals at P35 in order to evaluate growth velocity and the CI (Table 2). In line with previous studies by our group, by P2 no differences in BW were observed between sexes. However, at P35 IUGR females were significantly smaller than IUGR males (*p* < .05). The growth velocity in both CMO groups was similar and significantly slower than in the control group (*p* < .01, Table 2), and thus, the two CMO groups were smaller (21–26% respectively) than the controls at P35. Due to the allometric growth of the head and body, the CI at P35 was considerably smaller than at P2, and neonatal differences among groups were maintained along the postnatal development with ischemic and IUGR groups presenting a significantly higher CI than the controls (*p* < .01) (Table 2). A CI over 1.32 was observed in 93% and 87% of ischemic and IUGR pups, respectively, but only in 25% of the control pups. On analyzing the correlation between BW and the CI at P2, the rho value was −0.9599, suggesting an inverse correlation between BW and the CI. This relation was also inverse at P35, being −0.7144. There is no statistical significant correlation between Final EL and CI rho: −0.2049.

**Table 2:** Evolution of the anthropometric parameters in the experimental groups

	Control		CMO			
	Control (*N *= 68)		Ischemic (BW > 10%, *N *= 42)		IUGR (BW < 10%, *N *= 31)	
	Male (*N *= 37)	Female (*N *= 31)	Male (*N *= 20)	Female (*N *= 22)	Male (*N *= 12)	Female (*N *= 19)
Weight (g) at day 2	6.35 ± 0.79 [5.07–7.56]	5.91 ± 0.52 [5.30–6.83]	6.3 ± 0.43 [5.12–6.51]	5.83 ± 0.44 [5.58–6.73]	**4.39 ± 0.50** **,## **[4**.**12**–**4**.**87]**	**3**.**32 ± 0.648** **,## **[3**.**43**–**4**.**9]**

&	Male (*N *= 7)	Female (*N *= 9)	Male (*N *= 6)	Female (*N *= 9)	Male (*N *= 8)	Female (*N *= 6)
Weight (g) at day 35	144.09 ± 17.5	130.2 ± 9.3	**118.10 ± 14.22** **	**105.70 ± 15.99** **	**108.44 ± 14.50** **	**91.02 ± 16.14** §,**
Brain weight (g) at day 35	1.65 ± 0.12	1.60 ± 0.08	1.70 ± 0.08	**1.60 ± 0.07** §	**1.54 ± 0.10** *, ##	**1.47 ± 0.04** **,##
Growth velocity (g/day)	4.17 ± 0.50	3.75 ± 0.27	**3.40 ± 0.42** **	**3.02 ± 0.50** **	**3.15 ± 0.44** **	**2.63 ± 0.49** **,§
Cephalization index at day 35	1.16 ± 0.12	1.23 ± 0.10	**1.46 ± 0.15** **	**1.55 ± 0.24** *	**1.44 ± 0.12** **	**1.65 ± 0.29** §,**

IUGR, Intrauterine growth restriction; BW*,* Birth weight. In the table evolution of some antthropometrical parameters is presented.

Each group belongs to at least to three different litters. Data are indicated as mean ± SD.

&: These parameters are obtained from those animals used for protein extraction.

Significant differences (LSD test) compared to control **p < .01, *p < .05; to ischemic ^#^ *p* < .05; to male ^§^ *p* < .05.

### 3.2 Effect of utero‐placental insufficiency in spatial memory performance

**Figure 2:**
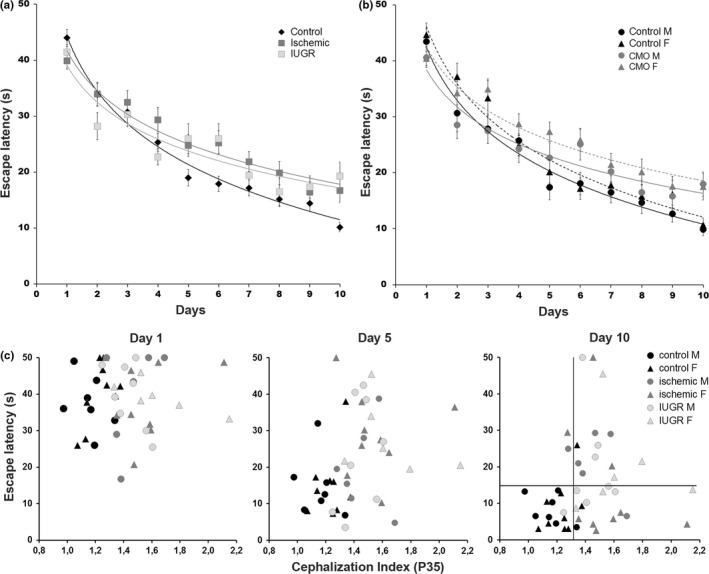
Learning progression and correlation between escape latency and the cephalization index (CI). (a) Plot representing the average escape latency in four trials performed each day by the controls and the ischemic and Intrauterine growth restriction (IUGR) groups (*n *= 55, *n *= 34, *N *= 22, respectively, with similar representation of both genders) during the 10 days of the study. Learning progression fit to logarithmic curves and ANOVA analysis revealed significant differences in the shape of the curve and the final scores between the controls and the IUGR and ischemic rats (*p* < .001), with no significant differences between IUGR and ischemic rats. (b) Plot representing the average escape latency for the same animals as in (a), but segregated by gender and distributed into controls and CMO (IUGR + ischemic) experimental groups. Multifactor ANOVA and LSD analysis revealed significant differences in the shape of the learning curves between males and females (*p* < .01 LSD test), and between their corresponding controls and CMO groups (males *p* < .05, females *p* < .01, LSD test). (c) Dispersion plots represent the average escape latency of each individual (control male = 7, control female = 9; ischemic male = 6; ischemic female = 9; IUGR male = 8; IUGR female = 6) with respect to its CI on three representative days of the study. Vertical and horizontal lines on day 10 indicate the 75th percentile of the controls (CI = 1.32; escape latency [EL] = 17.7 s). Error bars in a and b represent the standard error of the mean

The MWT modified for young animals was used to evaluate learning and spatial memory. The EL of each animal was recorded four times a day, for 10 consecutive days. The results were analyzed by multivariate ANOVA with respect to trial day, experimental group and gender, with all three parameters significantly affecting the learning process (*p* = .00001, *p* = .0008 and *p* = .0016, respectively). The performance of the controls, and the ischemic and IUGR groups improved over time (*p* < .01), but significant differences were observed in their learning progression (Figure 2a). Along the learning process, control rats were better (shorter EL) than ischemic and IUGR rats (*p* < .01 and *p* < .05, respectively). No significant differences were found between the ischemic and IUGR groups (*p* = .672). Final the EL values of the ischemic and IUGR animals remained significantly higher than those of the controls (controls: 10.2 ± 6.3 s; ischemic: 16.7 ± 12.9 s *p* < .05; IUGR: 19.3 ± 12.3 s, *p* < .01; mean ± SD).

We considered gender as a relevant factor in this analysis. However, no significant differences were found in the learning process between the ischemic and IUGR groups. Therefore, we clustered the two CMO groups together and repeated the analysis segregating the controls versus the CMO groups by sex (Figure 2b). The learning curves showed significant differences according to gender and the CMO procedure (*p* = .002 and *p* = .0034, respectively). Despite intergroup differences in the learning curves, the males and females in the different groups achieved similar final EL scores (control: ♂ 9.9 ± 5.9 s; ♀ 10.7 ± 6.6 s; CMO: ♂ 18.0 ± 10.9 s; ♀ 17.5 ± 13.2 s; mean ± SD). The CMO procedure affected both genders similarly (*p* = .6290), and the scores were significantly poorer than their respective controls (♂*p* = .01 and ♀ *p* = .05 LSD test).

Anthropometric parameters (Table 2), including body and brain weight, growth velocity and the CI were analyzed as possible covariables in the learning process. The learning process showed a significant association with the CI (males, *p* = .0039; females, *p* = .0240) but not with BW (males, *p* = .671; females, *p* = .469). Surprisingly, there was no correlation between the CI and MWT performance (*p* = .213). Independently of the experimental group, CI values above 1.32 were associated with an increased risk of poor spatial memory performance (EL below the 25th percentile, estimated in the control group), ranging from 33% in controls (1 of 3) to 58% in pups from CMO‐operated mothers (15 of 26) (Figure 2c).

These results suggest that a high CI associated with utero‐placental insufficiency predisposed the pups studied to learning and memory deficits.

### 3.3 Effect of utero‐placental insufficiency on cell number and synaptic protein expression in the hippocampus

**Figure 3:**
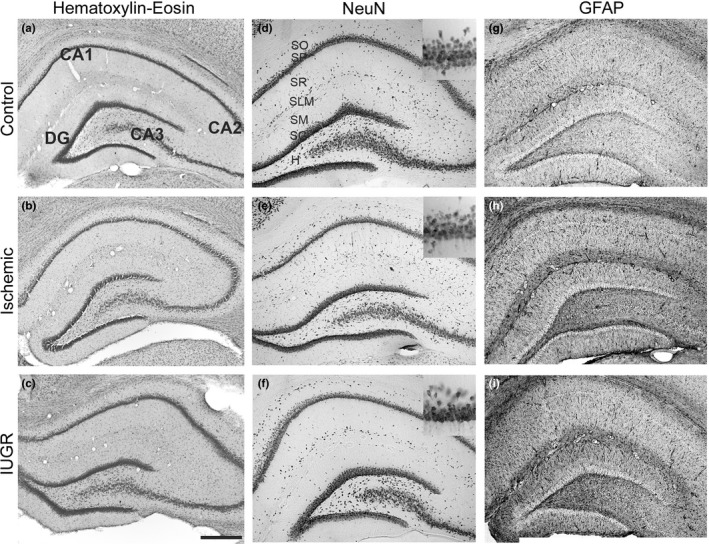
Hippocampus histology. Coronal hippocampal sections of control (a, d, g,) ischemic (b, e, h) and Intrauterine growth restriction (IUGR) (c, f, i) rats at P35. (a–c) Nissl stained; d–f) immunostained against NeuN, inset correspond to a higher magnification of the CA1 pyramidal layer; (g–i) GFAP immunostained. CA1, CA2, CA3 hippocampal fields; DG dentate gyrus; H, hilus; SO, stratum oriens; SP, stratum pyramidale; SR, stratum radiatum; SG, stratum granulosum; SLM, stratum lacunosum‐moleculare; SM, stratum moleculare; Scale bar: 150 μm

At P35, the hippocampus of ischemic and IUGR rats were normal without gross histopathological differences in H‐E (Figure 3a–c) NeuN (Figure 3d–f), and with mild gliosis observed in GFAP staining (Figure 3g–i). However, as reductions in the number of hippocampal cells have been described in other IUGR animal models (Mallard et al., 2000), we performed an in depth analysis of the impact of CMO‐induced utero‐placental insufficiency on the number of neurons immunolabeled with NeuN in the pyramidal layer of the CA1 subfield of the hippocampus (*n *= 3 from each experimental group). A small, albeit statistically significant, difference was found in the total number of neurons in the CA1 pyramidal layer in the ischemic and IUGR groups compared to controls (control = 65 ± 9; ischemic = 54 ± 7, *p* < .001; IUGR = 48 ± 4; mean ± SD, *p* < .001).

**Figure 4:**
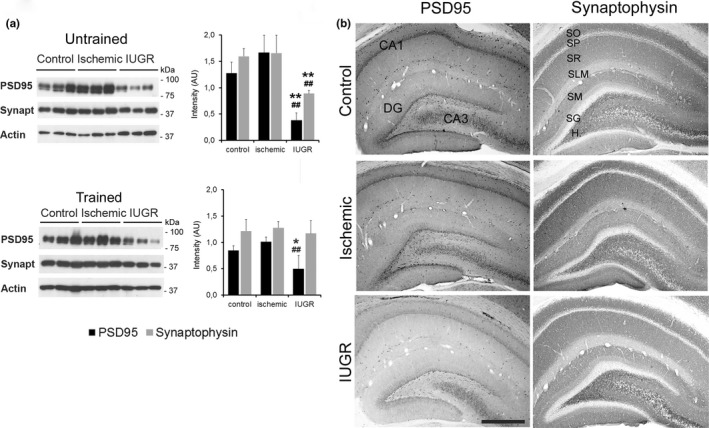
Distribution of synaptic proteins in the hippocampus. (a) Western blot of PSD95, synaptophysin and SNAP25 proteins in hippocampal tissue from controls, and the ischemic and Intrauterine growth restriction (IUGR) groups of untrained and water maze trained rats. Actin was used as the protein loading control. PSD95 and synaptophysin expression were significantly reduced in the untrained IUGR group, whereas only PSD95 expression was reduced after training. (b) Photomicrographs of coronal sections of the hippocampus of trained control, ischemic and IUGR rats immunostaining of postsynaptic protein PSD95, and presynaptic protein synaptophysin. CA1, CA3, hippocampal subfields; DG, dentate gyrus; H, hilus; SO, stratum oriens; SP, stratum pyramidale; SR, stratum radiatum; SG, stratum granulosum; SLM, stratum lacunosum‐moleculare; SM, stratum moleculare; *p < .05 respect control, ##*p* < .01 respect ischemic; LSD test. Scale bar: 185 μm

To evaluate the integrity of the functional synaptic machinery in the hippocampus, total protein content, and the distribution of two specific synaptic proteins, postsynaptic density protein 95 (PSD95) and synaptophysin, were analyzed by WB and immunofluorescence imaging (Figure 4a, b). PSD95 is a scaffolding protein involved in glutamate receptor clustering and postsynaptic intracellular signaling (Elias & Nicoll, 2007), and synaptophysin is an integral membrane vesicular synaptic glycoprotein implicated in vesicle biogenesis and recycling (Valtorta, Pennuto, Bonanomi, & Benfenati, 2004). WB analyses revealed that in both trained and untrained animals the total amount of PSD95 protein was significantly reduced in the hippocampus of IUGR animals compared to the control and ischemic groups. In contrast, PSD95 protein content tended to increase, albeit not significantly, in ischemic animals. A significant reduction was also found in the total amount of synaptophysin protein in the hippocampus of untrained IUGR animals compared to the control and ischemic groups. However, the total amount of synaptophysin protein was normalized in the hippocampus of IUGR rats after water maze training (Figure 4a). Immunohistochemistry analysis of the hippocampus of trained rats revealed a similar laminar distribution of PSD95 and synaptophysin staining in the three groups (Figure 4b). However, in line with the reduction in PSD95 protein observed in WB, the intensity of PSD95 immunostaining was reduced in the hippocampus of the IUGR group.

## 4 Discussion

The main findings of this study suggest that neurodevelopmental alterations affect males and females similarly and are not only associated with BW but also with an abnormally high CI and altered synaptic machinery in the hippocampus.

IUGR is one of the most frequent and complex conditions in modern obstetrics, being mainly produced by placental insufficiency and undernourishment (Goldenberg et al., 1989; Maulik, 2006). The incidence of IUGR has been on the rise worldwide in the last decades (Romo et al., 2009). The most frequently studied long‐term repercussions of IUGR are those involving metabolic programming, which lead to the so‐called metabolic syndrome that is associated with a high risk of developing hypertension, diabetes, and cardiovascular disease. Nonetheless, longitudinal studies have also revealed a high incidence of learning, school and behavioral problems in IUGR babies compared with babies born at the same gestational age but with an adequate BW (Leitner et al., 2007; Nardozza et al., 2012). It is important to note that the diagnosis of true IUGR remains a challenge in clinical practice since children <10th percentile in size are not always pathological, and some children with appropriate weight for their gestational age might not have achieved their target growth (Campbell et al., 2012; Zhang et al., 2010). In particular, the underlying neuropathology of cognitive and behavioral deficits associated with IUGR is largely unknown, and the identification of children at risk of poor neurological outcome is very difficult at early ages (Leitner et al., 2007; Streimish et al., 2012). IUGR fetuses develop some hemodynamic adaptations during fetal life, redistributing blood flow to vital organs such as the heart and brain. In these babies, the size of the head is relatively spared with respect to the growth of the body, resulting in an abnormally high CI. Although this brain sparing effect was initially considered to be a protective mechanism, it is now largely considered to be an early indicator of brain distress (Hernandez‐Andrade, Serralde, & Cruz‐Martinez, 2012; Yanney & Marlow, 2004) associated with an increased risk of altered neurological outcome (Figueras et al., 2011; Geva et al., 2006). Consequently, the CI has been proposed as a clinical parameter for the early detection of babies at risk of abnormal neurodevelopment (Fattal‐Valevski et al., 2009; Roza et al., 2008).

To understand the correlation between learning deficits and the morphological, cellular and molecular alterations developed in IUGR patients, many animal models have been developed. Rodents (mice, rats, and guinea pigs) are the most commonly used animal models to asses alterations associated with IUGR due to the high number of functional tests available that allow the evaluation of neurological outcome.

In the present work, we used a model of chronic utero‐placental insufficiency induced by the CMO procedure at E17. This model produces relatively uniform ischemia throughout the uterus and results in a 42% survival rate and IUGR with a significant brain sparing effect in the pups (Camprubi et al., 2009). As in other vascular models performed at day 17 of gestation (Huizinga et al., 2004) and at E18 (Jansson & Lambert, 1999; Ogata, Bussey, LaBarbera, & Finley, 1985), animals born to CMO mothers did not achieve catch up growth. Simmons, however, reported catch up growth in animals obtained from operated mothers at day E19 (Simmons et al., 2001).

To our knowledge, this modified version of the CI using the brain weight has not been used in other animal models. However, in a rabbit model created by Eixarch and colleges, the CI was also higher in IUGR, being 3.19 versus 2.5 in control animals and suggesting that the intrauterine process of growth restriction is similar (Eixarch et al., 2009).

To analyze differences in learning and memory processes between severely and mildly growth restricted rats, pups born to CMO‐operated mothers were divided according their BW into IUGR (<the 10th percentile), and ischemic, BW (>the 10th percentile) and within the normal limits. Despite the different BWs, the two CMO groups showed a significantly higher CI at birth and a lower growth velocity compared to controls thereby reflecting the absence of catch up. The brain sparing effect observed in CMO pups at birth (higher CI) was maintained throughout postnatal development, at least until day 35.

Learning evolution was analyzed by the MWT, a specific hippocampal‐related spatial memory test, and was found to be similar in the CMO groups. This test has the advantages of rapid acquisition; performance is less influenced by BW and does not require food or water deprivation as in other tests and could be a drawback in rapidly growing postweaning rats (Barrow & Leconte, 1996; Ehman & Moser, 2006). The early postweaning period in rats (P25‐P35) was chosen because this period represents a milestone in the progression to adult function in mammals and constitutes an essential step in learning performance in many tests (Ehman & Moser, 2006).

In general, learning curves were slightly different between males and females, although both genders achieved similar final scores according to their experimental group. Although sex differences have been suggested in the outcomes of animals subjected to neonatal hypoxia‐ischemia (Smith, Alexander, Rosenkrantz, Sadek, & Fitch, 2014), this is not supported by our data and water maze performance was affected similarly by the CMO procedure in both sexes. IUGR and ischemic animals of both sexes needed more time to learn the same task than their respective controls, and although their performance improved over time, their final scores remained slightly lower.

Our analysis also showed a correlation between CI > 1.32 (corresponding with the 75th percentile of the control group) and poor spatial memory (below the 25th percentile of the control group), although above this threshold, the largest CIs at P35 were not necessarily associated with the poorest scores. One potential explanation for this lack of linear correlation between CI and spatial memory performance may be that, with increasing severity of utero‐placental dysfunction and nutrient deprivation, critical adjustments in fetal brain metabolism might impair postnatal growth and neurodevelopmental outcome, masking the initial brain sparing effect and the increase in the CI. Although further studies and a systematic anthropometric analysis of individuals are needed to address this question, a recent systematic review of animal models that used uterine artery ligation to induce IUGR reached similar conclusions. Fetuses with growth restriction demonstrate a reduction in BW with relative sparing of brain mass and deficits in many neuroconductual tests (Basilious, Yager, & Fehlings, 2015).

Moreover, the functional alterations described in CMO animals in this study are in line with those described by Leitner and colleagues in a cohort of IUGR babies. These authors reported that IUGR babies present alterations in spatial orientation and spatial memory (Leitner, Heldman, Harel, & Pick, 2005). In addition, typical hippocampal deficits, such as executive short‐term memory deficits characteristic of anterior hippocampal‐prefrontal network alteration have also been reported in IUGR babies (Geva et al., 2006). The similar poor spatial memory performance shown by ischemic and IUGR rats might be related to their parallel anthropometric evolution, as described in a cohort of IUGR patients, in whom the presence of catch up growth was related to better neurological prognosis and an increased CI with learning and memory disabilities (Fattal‐Valevski et al., 2009; Leitner et al., 2007). Our data suggest that in cases in which utero‐placental insufficiency is suspected, a higher CI (estimated to be above the 75th percentile) maintained during childhood could be a better indicator of suboptimal intrauterine growth than only low BW. Moreover, a high CI value could also be useful as a marker of increased risk of cognitive and neurobehavioral deficits.

Spatial memory plays an important role in the consolidation of learning and is a major part of what is known as explicit memory or conscious memory of events that have occurred in the external world. In mammals, this process takes place in the CA1 region of the hippocampus, and the underlying cellular mechanism is a form of synaptic plasticity known as long‐term potentiation or LTP (Vann & Albasser, 2011), that requires Ca^+2^ signaling, gene expression changes and protein synthesis (Morgado‐Bernal, 2011). The neuropathological bases of learning and memory alterations in IUGR babies are not clear, but there is consensus that within the brain the hippocampus is one of the areas most susceptible to IUGR and hypoxic damage (Fung et al., 2012; Lodygensky et al., 2008; Mallard et al., 2000), which particularly affects CA1 pyramidal neurons in the hippocampus (Kovalenko et al., 2006; Lister et al., 2005). In this study, the ischemic and IUGR CMO groups presented a reduction in CA1 neuronal number, mild gliosis and paradoxical changes in PSD95 and synaptophysin expression. PSD95 is a neuronal protein anchored in the postsynaptic compartment of the glutamatergic neurons, where it acts as a scaffolding protein necessary for AMPAR and NMDAR glutamate receptor trafficking and transsynaptic signaling, thereby modulating excitatory synaptic strength, excitatory/inhibitory balance, and plasticity (Elias & Nicoll, 2007; Keith & El‐Husseini, 2008). Abnormally reduced PSD95 expression impairs activity‐dependent synaptic stabilization (Ehrlich, Klein, Rumpel, & Malinow, 2007), whereas PSD95 overexpression increases the excitatory to inhibitory synapses ratio and alters dendritic arbor development (Bustos et al., 2014; Prange, Wong, Gerrow, Wang, & El‐Husseini, 2004). PSD95 was dramatically reduced in the hippocampus of IUGR rats, similar to what has been described in animals exposed to perinatal hypoxia which also displayed impaired hippocampal maturation and deficits in spatial learning and memory (Chen et al., 2006, 2007). Abnormal PSD95 expression has also been associated with autism spectrum disorders in humans and animal models (Keith & El‐Husseini, 2008; Tsai et al., 2012), and these disorders have also been related to IUGR (Leonard et al., 2008; Walker et al., 2015). Presynaptic machinery is also affected by utero‐placental insufficiency and is shown by reduced protein levels of synaptophysin in the hippocampus of naive IUGR rats and the recovery of normal levels after training. Synaptophysin is a presynaptic protein involved in synaptic vesicle biogenesis and trafficking that increases in parallel with the formation of synapses and is frequently used as an indicator of total synaptic vesicles pools (Valtorta et al., 2004). It is known that positive stimuli such as physical exercise and environmental enrichment increase synaptic proteins expression and normalize interneuron development, and are associated with improved synaptic plasticity and cognitive function (Abel & Rissman, 2013; Chen, Chen, Lei, & Wang, 1998; Chen et al., 2006; Hu, Ying, Gomez‐Pinilla, & Frautschy, 2009; Komitova et al., 2013). Therefore, it is possible that exercise inherent to water maze training might account for the synaptophysin normalization in IUGR rats after training such as what was observed in this study, and suggests that environmental management might reverse some of the synaptic changes associated with prenatal growth restriction postnatally.

## 5 Conclusions

In this study, a murine model was used to determine part of the biological mechanisms of learning and memory disorders related to IUGR. Although interspecific differences in fetal response to placental insufficiency need to be addressed, the translation of these data to humans suggests that in addition to severely affected IUGR babies, babies with a normal BW but with intrauterine Doppler alterations or clinical indices of placental insufficiency and an abnormal CI (as in the ischemic group in this study) should also be closely followed up during their first years of life.

The CI is a simple easy measurement to standardize. The combination of an abnormally high CI at birth and the absence of catch up growth during the postnatal period could be an early detection criterion for the risk of long‐term neurobehavioral deficits. Moreover, as some of the synaptic deficits associated with placental insufficiency could be reverted postnatally, early detection of babies at risk could allow referral to appropriate management programs, including early stimulation and physical training, which can dramatically improve long‐term neurobehavioral outcome, decreasing or even preventing learning and memory disabilities in childhood and adolescence.
